# Identification of a novel homozygous *SLC13A5* nonstop mutation in a Chinese family with epileptic encephalopathy and developmental delay

**DOI:** 10.3389/fgene.2025.1474390

**Published:** 2025-04-17

**Authors:** Hua He, Lijuan Long, Manling Tang, Qiang Xu, Shengwu Duan, Ge Chen, Yan Zhao, Qiongfang Wu, Jia Chen

**Affiliations:** ^1^ Laboratory Medicine Center, Zhuzhou Hospital Affiliated to Xiangya School of Medicine, Central South University, Zhuzhou, China; ^2^ Department of Critical Care Medicine, Zhuzhou Hospital Affiliated to Xiangya School of Medicine, Central South University, Zhuzhou, China; ^3^ Reproductive Medicine Center, Jiangxi Maternal and Child Health Hospital, Nanchang, China; ^4^ Department of Radiology, Zhuzhou Hospital Affiliated to Xiangya School of Medicine, Central South University, Zhuzhou, China; ^5^ Central Laboratory, Jiangxi Maternal and Child Health Hospital, Nanchang, China

**Keywords:** developmental and epileptic encephalopathy, SLC13A5, whole exome sequencing, homozygous, nonstop mutation

## Abstract

**Introduction:**

Biallelic loss-of-function variants in the *SLC13A5* (solute carrier family 13, member 5) gene are responsible for autosomal recessive developmental and epileptic encephalopathy 25 with amelogenesis imperfecta (DEE25). Until now, no pathogenic variants of *SLC13A5* has been reported among the Chinese population.

**Methods:**

A Chinese Han pediatric patient with epilepsy and global developmental delay was described in this study. Trio-whole exome sequencing (WES) including the patient and her parents was performed to determine the genetic basis of the phenotype. Potential pathogenic variants were subsequently confirmed by Sanger sequencing. Additionally, we conducted an extensive review of the literature regarding *SLC13A5* variants to analyze their associated phenotypic characteristics.

**Results:**

Trio-WES revealed a novel homozygous variant c.1705T>G in *SLC13A5* associated with clinical manifestations in the proband. The variant was also detected in her parents and unaffected sister, who were both heterozygous carriers. The variant is a nonstop substitution that is predicted to extend the SLC13A5 protein by 174 amino acids (p.569Gluext174). Analysis of previously published cases indicated that *SLC13A5* patient in our study exhibited overlapping symptoms.

**Discussion:**

We identified a novel homozygous nonstop mutation in the *SLC13A5* gene of a Chinese patient with DEE25. This study expands the mutation spectrum of *SLC13A5* and will have significant implications for the proband’s family in terms of medical management and genetic counseling.

## 1 Introduction

Developmental and epileptic encephalopathies (DEEs) are a group of clinically and genetically heterogeneous neurological disorders characterized by frequent epileptic activity and developmental impairment (Guerrini et al., 2023). DEEs represent the most severe forms of epilepsy, typically manifesting in neonates or children (Scheffer et al., 2021). Genetic factors play a significant role in the development of DEEs. To date, over 100 genes have been associated with DEEs due to rapid advances in diagnostic tools, significantly enhancing our understanding of their molecular basis (McTague et al., 2016; Guerrini et al., 2023).

Autosomal recessive variants of the *SLC13A5* gene (MIM*608305) have been reported to cause developmental and epileptic encephalopathy 25 with amelogenesis imperfecta (DEE25, MIM #615905) in early infancy (Matricardi et al., 2020; Milosavljevic et al., 2022). The *SLC13A5* gene is located on chromosome 17p13.1 and consists of 12 exons (Inoue et al., 2002a). It encodes a plasma membrane sodium-dependent citrate carrier known as the Na + -coupled citrate (NaCT) transporter, which is highly expressed in the brain, teeth, liver, and testis (Inoue et al., 2002b). Neurons are incapable of *de novo* synthesis of tricarboxylic acid cycle intermediates, therefore, the uptake of intermediates like citrate is crucial for maintaining energy status and neurotransmitter production (Hardies et al., 2015; Henke et al., 2020). Biallelic mutations, whether homozygous or compound heterozygous, in the *SLC13A5* gene can impair citrate uptake, disrupting tricarboxylic acid cycle metabolism and resulting in neonatal epilepsy (Goodspeed et al., 2022). *SLC13A5* was first identified as a causative gene of DEE25 in 2014 (Thevenon et al., 2014). To date, over 40 pathogenic variants have been reported in the *SLC13A5* gene (Brown et al., 2021; Beltran, A.S., 2024). However, none of these variants have been reported in the Chinese population.

In our study, we identified a novel nonstop mutation in the *SLC13A5* gene in a Chinese family affected by infant epilepsy and developmental delay. The patient exhibited a range of clinical features, including dental anomalies, intellectual disability, motor difficulties, and speech impairments, alongside abnormalities detected through neuroimaging. We conducted a thorough literature review on *SLC13A5* variants to analyze their associated phenotypic characteristics.

## 2 Materials and methods

### 2.1 Whole-exome sequencing and analysis

Ethical approval for this study was granted by the Ethics Committee of Jiangxi Maternal and Child Health Hospital. Written informed consent was obtained from the parents of the patient. Genomic DNA was extracted from peripheral blood samples using the Gentra Puregene Blood Kit (Qiagen, Hilden, Germany) following the manufacturer’s protocol. The main steps of whole exome sequencing (WES) analysis were performed as previously described (Guo et al., 2018; Chen et al., 2020). Exomes were captured using the SureSelect XT Human All Exon V6 kit (Agilent Technologies, Santa Clara, CA, United States) and sequenced on an Illumina HiSeq sequencer (Illumina, San Diego, CA, United States). After quality control assessment with FastQC, reads were mapped to the UCSC RefSeq database hg19 human reference genome using BWA to remove duplications. Variant calling and annotation were conducted using GATK and ANNOVAR. Candidate mutations identified by WES were confirmed by Sanger sequencing. The pathogenicity of these candidate variants was assessed following the 2015 ACMG/AMP classification guidelines (Richards et al., 2015).

### 2.2 Protein predictions

The protein sequence of human SLC13A5 was retrieved from the UniProt database (https://www.uniprot.org/), and AlphaFold2, an online protein structure prediction tool, was utilized for modeling SLC13A5 structure. All structural figures were generated using PyMol.

## 3 Results

### 3.1 Clinical features of the patient

**FIGURE 1:**
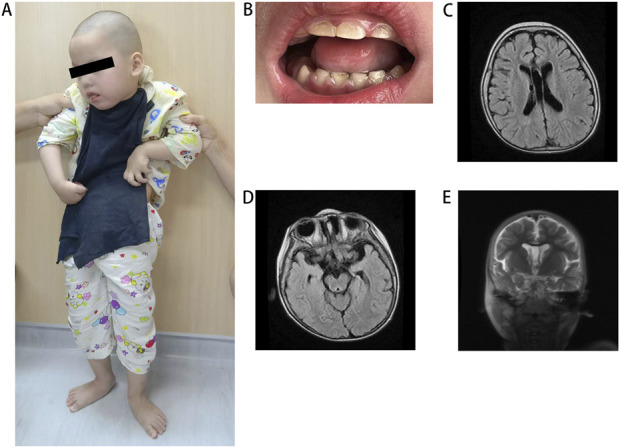
Clinical features of the patient. **(A)** The patient at the standing position. **(B)** Illustrative picture of teeth hypodontia and amelogenesis imperfecta in patient. **(C)** MRI shows white matter demyelination, **(D)** enlargement of the temporal horn of the lateral ventricle and **(D, E)** atrophy of the hippocampus and parahippocampal gyrus. MRI, magnetic resonance imaging.

The proband was a 5-year-old girl born to physically healthy via cesarean section at term. The parents were non-consanguineous and from different cities in Jiangxi province. The proband experienced a multifocal status epilepticus characterized by long-lasting focal seizures the day after birth. During her first year, seizures were occurred approximately every 3–4 months. Following treatment with antiseizure medications valproic acid and levetiracetam, seizure frequency significantly decreased. She exhibited profound intellectual disability and severe motor impairment, presenting with autistic-like behavior characterized by lack of eye contact, inability to speak, and disinterest in surroundings. She required support to stand or sit due to severe muscle tone, particularly in both upper limbs (Figure 1A). Dental hypoplasia and amelogenesis imperfecta were noted (Figure 1B). Brain MRI revealed demyelination in the white matter (Figure 1C), enlargement of the temporal horn of the lateral ventricle, and atrophy of the hippocampus and parahippocampal gyrus (Figures 1D, E). The patient had a healthy 12-year-old sister and another sister who exhibited similar symptoms and died from seizures at 3 months of age, neither had undergone genetic testing.

### 3.2 Identification of a nonstop *SLC13A5* variant

**FIGURE 2:**
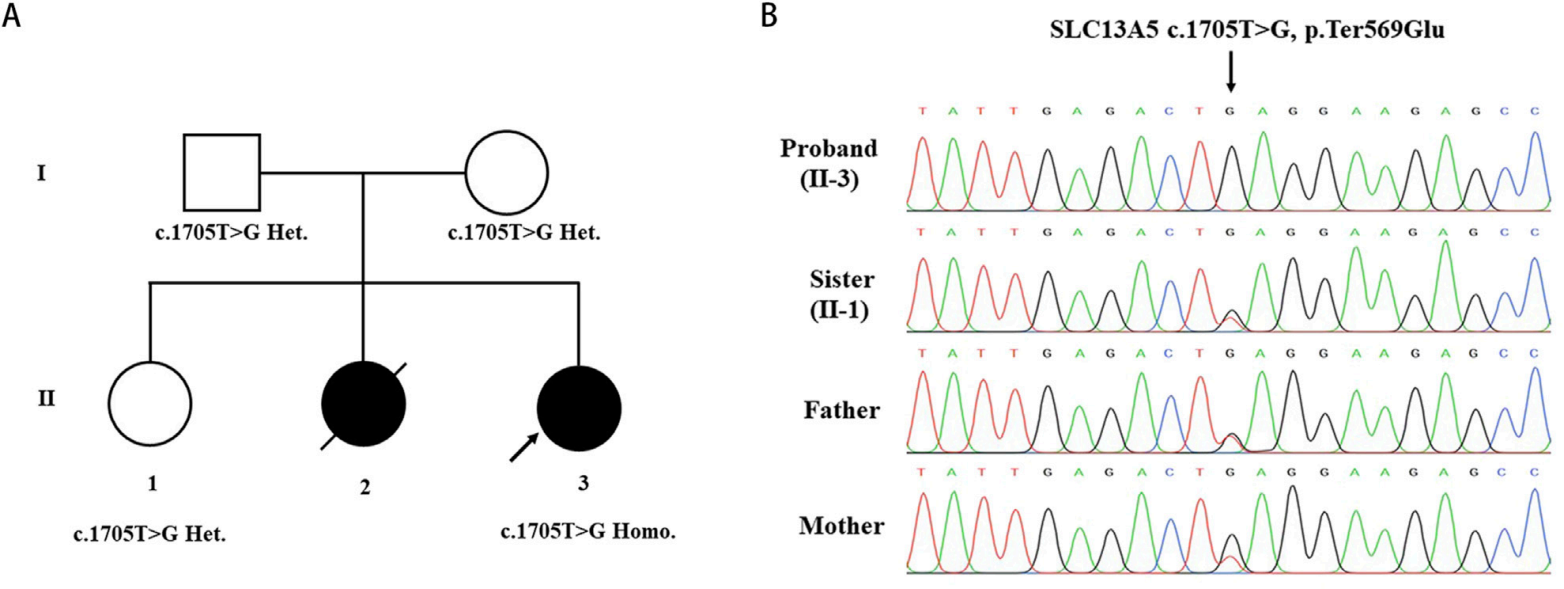
Identification of the SLC13A5 Variant. **(A)** Pedigree of the investigated family. **(B)** Sanger sequencing show homozygous mutation in the proband(II-3), heterozygous mutation in sister (II-1) and heterozygous mutation in parents. Arrow indicated the nonstop mutation changing the TAG stop codon in GAG.

Whole-exome sequencing analyses were conducted on the patient and her parents to identify the genetic basis of her condition. A homozygous variant (c.1705T>G) in the *SLC13A5* gene (NM_177,550.5) was identified as contributing to the patient’s symptoms. Sanger sequencing confirmed the homozygous mutation in the patient and revealed that her parents and unaffected sister were heterozygous carriers of the c.1705T>G (p.569Gluext174) mutation (Figure 2A, B). This variant has not been previously reported in the literature and is absent from the gnomAD, HGMD, and ClinVar databases, suggesting it is a novel causative variation for DEE25. According to the 2015 ACMG guidelines [15], the pathogenicity of the c.1705T>G variant was classified based on criteria PM2_Supporting + PM4_Very strong, indicating it is likely pathogenic. No additional variants in genes associated with epilepsy and/or dental abnormalities were identified in the proband.

### 3.3 Structural modelling of the *SLC13A5* Variant

**FIGURE 3:**
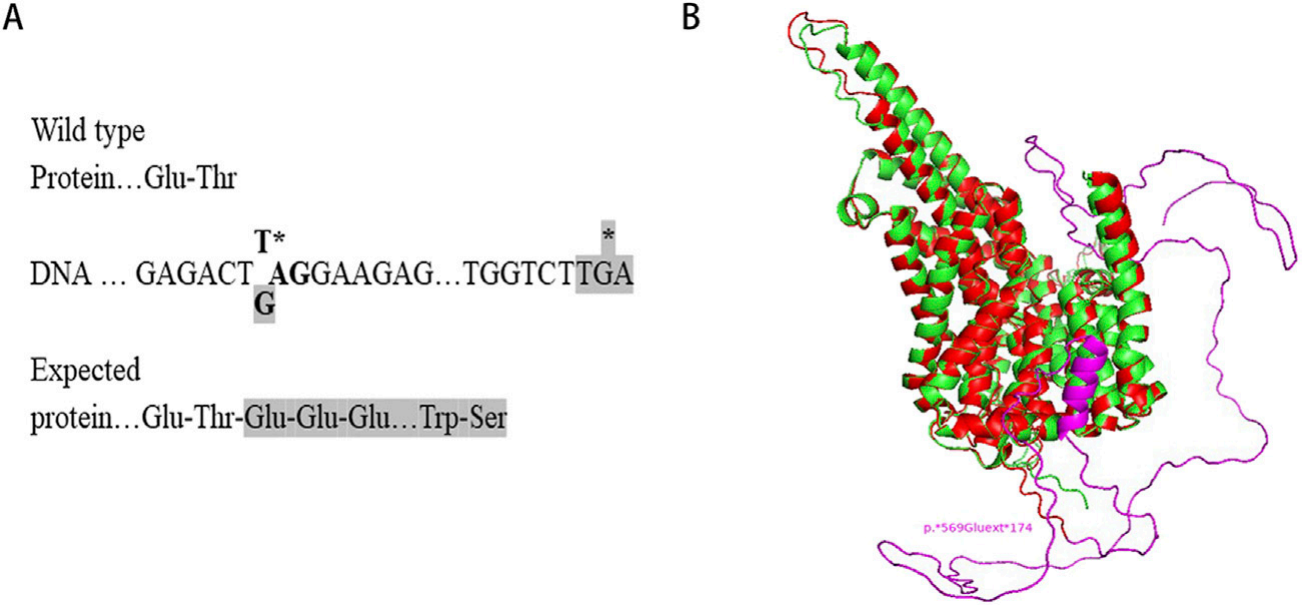
Structural modelling of the mutant SLC13A5. **(A)** Protein expressed from the mutant allele is C-terminally extended. **(B)** Structural modeling of SLC13A5 protein by AlphaFold2. The p. Ter569Glu Variant, wild type, and extended portion are shown in red, green, and purple, respectively. Models are visualized in PyMOL.

Sequence analysis revealed that the c.1705T>G variant identified in this study changes the TAG stop codon of the *SLC13A5* gene to a glutamic acid-encoding GAG codon. Downstream at position c.*520_522, there is another TGA stop codon, resulting in the expression of a chimeric protein composed of the regular 568 SLC13A5 residues and an additional 174-residue C-terminal extension (p.569Gluext174) (Figure 3A). Structural modeling indicated that the substitution of the stop codon to glutamic acid created an α-helical domain and random coils that were absent in the wild-type protein (Figure 3B).

## 4 Discussion

Biallelic loss-of-function mutations in the *SLC13A5* gene typically lead to DEE25, characterized by neurological manifestations such as epilepsy and psychomotor developmental delay (Alsemari et al., 2024). In this study, we identified a novel *SLC13A5* nonstop variant (c.1705T>G) in a Chinese girl who began experiencing seizures shortly after birth. The phenotypic spectrum observed in our patient overlaps with previously reported cases, including early onset seizures, profound intellectual disability, global developmental delay, and dental abnormalities (Supplementary Table 1). Additionally, our patient exhibited auxiliary features such as brain MRI abnormalities and hypertonia. According to the literature, neuroimaging abnormalities are present in approximately one-third of *SLC13A5*-related DEE cases, with white matter abnormalities being the most common (Whitney et al., 2023). The affected patients may initially present with infantile hypotonia and later develop increased muscle tone, pyramidal signs, spasticity, dystonia, and/or ataxia. This range of symptoms reflects the clinical heterogeneity of the disease (Arvio and Lähdetie, 2020).

From the literature review (Supplementary Table 1), most *SLC13A5* variants are missense mutations, though a smaller number of frameshift, splicing, insertion, and intronic mutations have also been reported. These variants are typically either homozygous or compound heterozygous and follow an autosomal recessive inheritance pattern. Patients with these mutations exhibit developmental impairments of varying severity, often accompanied by a range of neurological signs and symptoms, including axial hypotonia, peripheral hypertonia, dystonia, dyskinesia, spasticity, ataxia, choreoathetosis, and microcephaly. Current clinical and genetic data do not show consistent genotype-phenotype correlations (Matricardi et al., 2020). Even within families with more than one affected child, there is significant phenotype heterogeneity among siblings with identical genotypes (Anselm et al., 2017; Arvio and Lähdetie, 2020; Matricardi et al., 2020).

The human SLC13A5 protein consists of 568 amino acids and is located at the plasma membrane, where it mediates the transport of citrate from extracellular environment into the cell (Inoue et al., 2002a). Citrate is crucial for maintaining cellular metabolic homeostasis, particularly in energy production and neurotransmitter synthesis within the central nervous system (CNS) (Li and Wang, 2021). Secondary structure predictions indicate that SLC13A5 is predominantly α-helical, comprising approximately 65.3% α-helices, including 11 transmembrane α-helices, with 33% in loops and less than 2% in β-strands (Jaramillo-Martinez et al., 2021). The protein forms a homodimer, with each protomer containing a scaffold domain and a transport domain (Sauer et al., 2021). Mutations in *SLC13A5* can disrupt citrate transport directly or affect protein expression and localization, depending on the position of the mutated residue (Jaramillo-Martinez et al., 2021; Beltran, 2024). Our case study presents a novel nonstop mutation that results in a C-terminally extended protein with an additional 174 residues (p.569Gluext174). Structural modeling revealed that this mutation introduces an α-helical domain and random coils not present in the wild-type protein. Functional research, such as citrate uptake assays, is needed to elucidate the underlying mechanisms of the mutation’s defects in SLC13A5-related epilepsy.

Nonstop variants, which convert stop codons into sense codons, are exceedingly rare mutations reported in various diseases (Hamby et al., 2011; Vaché et al., 2023), such as autosomal dominant Charcot-Marie-Tooth disease type 2 (Bock et al., 2018) and excessive hemorrhaging due to mutations in coagulation factor X (Ameri et al., 2007). When an alternative in-frame stop codon exists in the 3′untranslated region (UTR), translation can continue normally, resulting in the production of a C-terminally extended protein. The pathogenicity of these extra polypeptides has been investigated in a few cases. Studies have shown that these mutations can lead to altered subcellular localization in some instances (Sun et al., 2017) and accumulation of mutant proteins in others (Bock et al., 2018). These findings suggest that C-terminally extended proteins can confer a toxic gain-of-function, which contrasts with the loss-of-function effects typically seen in nonstop decay pathways. Further research is needed to fully understand the genetic mechanisms underlying C-terminal elongation in SLC13A5 and its implications for disease pathogenesis.

In summary, we identified the first *SLC13A5* mutation (c.1705T>G, p.569Gluext174) in a Chinese population. This nonstop substitution is rare and broadens the mutational spectrum of the *SLC13A5* gene. The discovery of this pathogenic variant will have significant implications for the proband’s family in terms of medical management and genetic counseling.

## Data Availability

The original contributions presented in the study are publicly available. This data can be found here: https://figshare.com/, https://doi.org/10.6084/m9.figshare.28795916.v1.
