# Supplementary material for: Identification of a novel homozygous SLC13A5 nonstop mutation in a Chinese family with epileptic encephalopathy and developmental delay
Source: Front Genet. 2025 Apr 17;16:1474390. doi: 10.3389/fgene.2025.1474390 (PMC12043571; doi:10.3389/fgene.2025.1474390)
Supplement: Supplementary file 1 [file Table1.docx]

**Table S1 SLC13A5 mutations and clinical features of the patients previously reported in the literature.**

| **Reference** | **Family** | **Mutation**  **Het/Hom** | **Sex/age**  **(years)** | **Age at first seizures** | **Brain MRI** | **EEG** | **Teeth** | **Main Symptom** |
| --- | --- | --- | --- | --- | --- | --- | --- | --- |
| Current report | F1 | c.1705T>G  (p.*569Gluext*174)  Hom | F/5 | 1 day | White matter demyelination, enlargement of the temporal horn of the lateral ventricle and atrophy of the hippocampus and parahippocampal gyrus | NA | Teeth hypoplasia and amelogenesis imperfecta | Profoundly ID, severe motor handicap, autistic, no eye contact, no speech, hypertonia |
| Thevenon et al., 2014 | F1 | c.655G>A (p.Gly219Arg)  c.680C>T (p.Thr227Met)  Het | M/14 | 30 hours | NA | Oscillating delta-theta waves that increased in amplitude, restricted to the temporal and occipital brain areas. | Widely spaced teeth | Profound psychomotor delay with no eye contact and global hypotonia, severe EE with no speech and no sitting position, no head control, no prehension |
|  |  |  | F/3 | 5 days | Normal | Rhythmic theta-delta focal discharges originating alternatively from both hemispheres, mostly in the left and right temporal regions | NA | Severe psychomotor delay, severe EE with no speech and no sitting position, proper eye contact, head control |
|  | F2 | c.1463T>C  (p.Leu488Pro)  Hom | F/10 | 1 day | NA | Asymmetrically slower on right side | Widely spaced teeth | Profound psychomotor delay, severe EE, poor eye contact and no sitting, mild perceptive deafness, smiling response, head control |
|  |  |  | M/5 | 8 hr | NA | NA | Hypodontia | Severe psychomotor delay, axial hypotonia and peripheral hypertonia with brisk tendon reflexes, severe EE, several words, walks with aids |
|  |  |  | M/3 | 1 day | NA | Continuous sequences of focal seizures involving temporal and occipital regions of both hemispheres alternatively | NA | Severe psychomotor delay, severe EE, no walking, several words, crawling |
|  |  |  | M/11 | 1 week | NA | NA | NA | Profound psychomotor delay, severe EE, proper eye contact, head control, no sitting |
|  |  |  | M/7 | 1 week | NA | NA | NA | Profound psychomotor delay, severe EE, proper eye contact, head control, no sitting, no speech |
|  | F3 | c.655G>A (p.Gly219Arg)  c.680C>T (p.Thr227Met)  Het | M/5 | 2 hours | NA | NA | NA | Severe pharmacoresistant EE associated with major developmental delay, eye contact, head control, no speech |
| Hardies et al., 2015 | F1 | c.1280C>T  (p.Ser427Leu)  Hom | M/13 | 1 day | Multiple punctuate haemorrhages white matter compatible with periventricular leukomalacia | Initially normal, then disontinuous with multifocal epileptiform discharges temporo-occipital regions | Hypoplasia | Severe ID, no speech,spastic tetraplegia, secondary microcephaly |
|  |  |  | M  Died at age 7y | 1 day | Periventricular leukomalacia | Epileptic discharges over bilateral central regions | Hypoplasia | Severe ID, speaks a few words, spastic tetraplegia, secondary microcephaly |
|  | F2 | c.1022G>A  (p.Trp341*)  c.1207_1217dup11  (p.Pro407Argfs*12)  Het | M/9 | 1 day | Normal | Initially normal, then focal right centro-occipital spikes and spike waves. | Hypoplasia | Severe ID, walks with support, speaks single words, pyramidal and extrapyramidal increased tonus with bilateral Babinski reflexes, choreoathetosis,  hypersalivation, strabism |
|  |  |  | F/7 | 1 day | Normal | Focal spike waves left centrooccipital | Hypoplasia | Mild-Moderate ID, walks unassisted, speaks short sentences, mild right hemiparesis, choreoathetosis |
|  | F3 | c.425C>T  (p.Thr142Met)  c.655G>A  (p.Gly219Arg)  Het | F/18 | 1 day | Normal | Normal interictal EEG | Hypoplasia | Mild-moderate ID with predominant speech deficits, coordination deficit, no clear ataxia |
|  |  |  | F/12 | 1 day | Normal | Multifocal spike waves (EEG review not possible) | Hypoplasia | Moderate ID with predominant speech deficits, coordination deficits, no clear ataxia |
|  |  |  | M/10 | 1 day | Normal | Multifocal spike waves (EEG review not possible | Hypoplasia | Severe ID with predominant speech deficits, autistic traits,moderate motor delay, ataxia |
|  | F4 | c.680C>T  (p.Thr227Met)  c.1570G>C  (p.Asp524His)  Het | F/4 | 1 day | Three small punctate white matter lesions, bilateral | Multifocal sharp slow waves. | Hypoplasia | Severe ID, head support, does not  walk nor speak, Generalized hypotonia, truncal ataxia |
| Klotz et al., 2016 | F1 | c. 655 G > A  (p. G219R)  c.1475 T > C  (p. L492P)  Het | F/11.8 | 1 week | Normal | Focal sharps, generalized spike-wave, normal background | Teeth hypoplasia | Motor and language delays, prolonged seizures, low muscle tone |
|  |  |  | M/2 | 1 week | Normal | Focal spikes, excess beta |  |  |
|  | F2 | c. 680 C > T  (p. T227M)  Hom | F/2.9 | 1 week | Normal | NA | Teeth hypoplasia +  Gingival hyperplasia | Motor and language delays, prolonged seizures |
|  | F3 | c. 245 A > G  (p. Y82C)  c. 655 G > A  (p. G219R)  Het | F/5.7 | 1 week | Focal frontal lobe  thickening | Right frontal spikes | Gingival hyperplasia | Motor and language delays, prolonged seizures, low muscle tone |
|  |  |  | M/8.7 | 1 week | Normal | Focal spikes associated  with sleep |  |  |
|  | F4 | c.511delG (DelG)  (p.E171SfsX16)  Het | F/9.6 | 1 week | Normal | Most recent EEG normal | NA | Motor and language delays, prolonged seizures |
|  | F5 | c.1276-1G > A  (Affect splice site)  c.103-1G>A  (& add intron**)  Het | F/18.7 | <1 year | Hyperintense foci in parietal white matter | Interictal epileptiform activity  higher in centrotemporal  regions, left > right,  especially during sleep | Teeth hypoplasia +  amelogenesis imperfecta | Motor and language delays, prolonged seizures |
|  |  |  | M/15.5 | 1 week | Normal | Irritability in right  centrotemporal and left  temporal areas | Amelogenesis imperfecta |  |
|  | F6 | c. 655 G > A  (p. G219R)  Hom | M/4 | 1 week | Focal loss of gray matter | Focal seizures | Normal | Motor and language delays, prolonged seizures, increased muscle tone |
| Weeke et al., 2017 | F1 | c.655G>A  (p.Gly219Arg)  c.1280C>T  (p.Ser427Leu)  Het | F/9 | 1 day | Extensive bilateral punctate white matter lesions, small intraventricular hemorrhage | Discontinuous normal voltage recovering | Teeth abnormalities | Cognitive Impairment, motor impairment, fever sensitivity |
|  |  |  | M  Post-mortem | 1 day | Suspected punctate white matter lesions | Discontinuous normal voltage recovering | NA  (Too young died) | NA(Died) |
|  | F2 | c.680C>T  (p.Thr227Met)  c.1280C>T  (p.Ser427Leu)  Het | M  Post-mortem | 1 day | Multiple bilateral punctate white matter lesions, intraventricular hemorrhage, cerebellar hemorrhage | Discontinuous normal voltage recovering | NA  (Too young died) | NA(Died) |
|  | F3 | c.655G>A  (p.Gly219Arg)  Hom | F/15 | 1 day | Extensive bilateral punctate white matter lesions | Discontinuous normal voltage recovering | NA  (Too young died) | NA(Died) |
|  |  |  | F/15 | 1 day | Five small punctate white matter lesions bilateral, delayed gyrification, cortical stroke | Discontinuous normal voltage recovering | NA  (Too young died) | NA(Died) |
| Schossig et al.,2017 | F1 | c.997C>T  (p.Arg333Ter)  Hom | M | 1 day | Normal | Epileptiform potentials with different foci, sometimes mixed with focal slowing | Hypoplastic, amelogenesis imperfecta with Yellowish to brownish | Severe ID, infantile hypotonia,  later spasticity, ataxia, dystonic gait,  strabismus |
|  |  |  | F | 7 months | Normal | Mmultifocal epileptiform potentials at the age of 7 months, monomorphic theta rhythms at the age of 2½ and later on generalised spike wave activity |  | Severe ID, infantile hypotonia, later ataxic dystonic gait, Horner syndrome |
|  | F2 | c.203C>A  (p.Pro68Gln)  c.434C>A  (p.Thr145Lys)  Het | F | 1 day | Normal | NA | Hypoplastic, amelogenesis imperfecta | Severe ID, spastic diplegia, pyramidal signs of the legs, cerebellar ataxia, strabismus |
|  |  |  | F | 2 months | Normal | NA | Amelogenesis imperfecta | Severe ID, pyramidal signs of the legs, cerebellar ataxia |
|  | F3 | c.103-1G>A  (p.? splicing Error)  c.1276-1G>A  (p.? splicing Error)  Het | F | 1.5 months | Normal | Hypsarrhythmia | Hypoplastic, amelogenesis imperfecta | Severe ID, hypotonia, ataxia |
|  |  |  | M | 1 day | Normal | Spiking activity in the frontotemporal region more frequent on the left. |  |  |
|  | F4 | c.680C>T  (p.Thr227Met)  Hom | F | 1 day | Mild atrophy of left  hemisphere | Frequent centro-parietal epileptiform activity with sharp waves, isolated spikes as well as spike waves. | Hypoplastic, amelogenesis imperfecta | Severe global developmental delay, marked hypotonia |
| Bainbridge et al., 2017 | F1 | c.997C>T  p.Arg333X  c.680C>T  (p.Tyr227Met)  Het | M/11 | 1 day | NA | NA | NA | Medically intractable epilepsy, normal growth parameters |
| Anselm et al., 2017 | F1 | c.655G>A  (p.Gly219Arg) c.245A>G (p.Tyr82Cys)  Het | M/8 | 1 day | Delayed myelination | Multifocal sharp waves | Altered enamel | Small stature, physical examination was normal, truncal hypotonia with mildly increased tone in his legs. |
|  |  |  | F/5 | 1 day | Blurring of the gray and white matter junction | Near continuous sharp waves in the right frontal area and continuous generalized slowing | Altered enamel | Severe motor delay, muscle tone is increased in both upper and lower extremities with severe truncal hypotonia, sociable |
| Alhakeem et al., 2018 | F1 | c.1227_1228insC  (p.(Ile410Hisfs*13)  Het | NA | 1 day | Posterior periventricular/peritrigone hyperintensity with reduction in the volume of white matter | Multifocal epileptiform discharges | NA | Psychomotor delay, can sit down, roll over |
|  |  |  | NA | 1 day | Unremarkable | Focal left temporal discharges | NA | Psychomotor delay, can sit down, roll over |
|  |  |  | NA | 1 day | Unremarkable | Many sharp wave and poly spikes in the left side | NA | Psychomotor delay, Hypotonic, sits without support |
| Costain et al., 2019 | F1 | c.716 + 5G > A  Hom | F/7 | 1 day | Normal | NA | NA | Global developmental delay, dyskinetic movements, microcephaly |
|  |  |  |  |  |  |  |  |  |
| Matricardi et al.,2020 | F1 | c.655G>A  (p.Gly219Arg)  c.1421C>T  (p.Pro474Leu)  Het | M/16 | 2 day | NA | NA | Tooth hypoplasia | Severe development delay, scoliosis, dystonia, tetraparesis |
|  | F2 | c.655G>A  (p.Gly219Arg)  del exons 1-5  (del exons 1-5)  Het | M/6 | 2 day | NA | NA | Tooth hypoplasia | Severe development delay, hypotonia, ataxia, brisk reflex, microcephaly |
|  | F3 | c.655G>A  (p.Gly219Arg)  c.1280C>T  (p.Ser427Leu)  Het | M/8 | 1 day | NA | NA | Tooth hypoplasia and hypodontia | Severe development delay, hypotonia |
|  | F4 | c.655G>A  (p.Gly219Arg)  c.943G>A  (p.Ala315Thr)  Het | F/5 | 1 day | NA | NA | NA | Normal development |
|  |  |  | F/3 | 1 day | NA | NA | NA | Normal development |
|  | F5 | c.997C>T  (p.Arg333*)  Hom | F/24 | 1 day | NA | NA | Tooth hypoplasia and hypodontia | Severe development delay, dyskinesias, tetraparesis |
|  |  |  | F/24 | 1 day | NA | NA | Tooth hypoplasia and hypodontia | Severe development delay, dyskinesias, tetraparesis |
|  | F6 | c.425C>T  (p.Thr142Met)  Hom | M/5 | 1 day | NA | NA | Tooth hypoplasia and hypodontia | Moderate development delay, ataxia, pyramidal signs |
|  |  |  | F/18 | 2 day | NA | NA | Tooth hypoplasia and hypodontia | Moderate development delay, axial hypotonia, ataxia, peripheral hypertonia, scoliosis |
|  | F7 | c.1475T>C  (p.Leu492Pro)  Hom | F/16 | 2 day | NA | NA | Tooth hypoplasia and hypodontia | Severe development delay, ataxia, hypotonia |
|  | F8 | c.369-2A>G  (—)  Hom | M/11 | 1 day | NA | NA | Tooth hypoplasia and hypodontia | Severe development delay, ataxia, dystonia, dyskinesias, delayed skeletal age |
| Arvio et al., 2020 | F1 | c.655G>A  (p. Gly219Arg)  Hom | F/56 | 1 day | NA | Right sided paroxysmal centro-parietal spikes | Lost all her teeth because of caries | Profoundly ID, severe motor handicap, squint, osteoporosis, no eye-contact, nor communication,cannot stand or sit unsupported, Increased muscle tone, short stature |
|  |  |  | M/54 | 1 day | NA | Cortical atrophy | Affected by strong abrasion and/or erosion | Profoundly ID, severe motor handicap, little communication. sit independently for a moment, walk with a walker, short stature |
| Jiang et al.,2021 | F1 | c.202C>T  (p.P68S)  c.429_c.437  delGCCGTGGTT  (p.A143_A146delinsA)  Het | M/3.5 | 14day | Normal | Focal spike wave | NA | Global Developmental delay and ID, hypotonia |
| Duan et al.,2021 | F1 | 88.5 Kb novel homozygous deletion involving SLC13A5 and XAF1 | M/2 | Shortly after birth | Nonspecific enlargement of subarachnoid spaces with normal cerebrum, cerebellum, and myelination for age. | Normal | Normal | Profound global developmental delay and ID, self-injurious behavior, hypertonia |
|  |  |  | M/15 | At birth | NA | NA | Hypoplastic enamel | Profound global developmental delay and ID, autistic behaviors, hypertonia, ataxic, short stature |
|  |  |  | M/11 | 3 day | NA | NA | Hypoplastic enamel | Profound global developmental delay and ID, mild autistic features, self-injurious behavior, mild hypertonia, ataxic |
| Nashabat et al.,2019 | F1 | c.1227dupC  (p.Ile410Hisfs*13)  Hom | F/7 | 2 day | Delayed myelination  for age, nonspecific  spectral pattern, and  microcephaly | Excessive beta activity (may be secondary to medications) Others normal, no epileptic  discharges | hypodontia | Delayed development, mild hypotonia, Microcephaly |
|  | F2 | c.655 G > A  (p.Gly219Arg)  Hom | M/5 | 2 day | Arachnoid cyst in the  right temporal lobe | Consistent with Lennox Gastaut syndrome (LGS). |  | Delayed development, hypotonia |
|  | F3 | c.231 + 2T > G  Hom | M/7 | Neonatal | Unremarkable | Multifocal |  | Profound global developmental delay and ID |
|  | F4 | c.785 T > C  (p.Leu262Pro)  Hom | F/12 | Neonatal | Unremarkable | Multifocal |  | Global developmental delay and ID |
|  | F5 | c.1227dupC  (p.Ile410Hisfs*13)  Hom | M/4 | Neonatal | Unremarkable | Multifocal |  | Global developmental delay and ID |
|  | F6 | c.1227_1228insC  (p.Ile410Hisfs*13)  Hom | M/10 | Neonatal | Unremarkable | Multifocal |  | Global developmental delay and ID |
|  | F7 | c.1227_1228insC  (p.Ile410Hisfs*13)  Hom | M/5 | Neonatal | Unremarkable | Multifocal |  | Global developmental delay and ID |
|  | F8 | c.1227_1228insC  (p.Ile410Hisfs*13)  Hom | M/11 | Neonatal | Unremarkable | Multifocal |  | Global developmental delay and ID |
|  | F9 | c.785 T > C  (p.Leu262Pro)  Hom | F/8 | 1 year | Unremarkable | Multifocal |  | Global developmental delay and ID |
|  | F10 | c.785 T > C  (p.Leu262Pro)  Hom | F/1 | Neonatal | Unremarkable | Epileptic Encephalopathy |  | Delayed development |
|  | F11 | c.1654 T > A  (p.Phe552Ile)  Hom | F/2 | Neonatal | Unremarkable | Multifocal |  | N/A |
| AlQudairy et al.,2023 | F1 | c.1227dupC  (p.I410H*13)  Hom | F/17 | 1 day | Normal | Intermittent slow activity | Hypodontia, teeth  hypoplasia, widely  spaced teeth,  gingival  hyperplasia | Global developmental delay and ID, motor difficulties, speech problems |
|  |  |  | F/14 | 1 day | Normal | Mild abnormalities are probably due to medication effect | Hypodontia,  teeth  hypoplasia,  widely spaced  teeth, gingival  hyperplasia | Global developmental delay, motor difficulties, speech problems, hypotonia |
|  | F2 | c.1227dupC  (p.I410H*13)  Hom | F/6 | 1 day | Unremarkable apart from mild enlargement of subarachnoid spaces and ventricular system | Focal epileptogenicity from  the left occipitotemporal  head region | Hypodontia, teeth  hypoplasia, widely  spaced teeth, gingival  hyperplasia | Global developmental delay, muscle  weakness, motor difficulties |
|  | F3 | c.1437 + 5G > A  Hom | F/28 | 2 day | Normal | The last EEG being normal. | Hypodontia, teeth hypoplasia, widely spaced teeth, gingival hyperplasia | Global developmental delay, muscle  weakness, motor difficulties, speak a few words, divergent squint |
|  |  |  | F/17 | Neonate | Focal cortical  dysplasia, minimal cortical thickening in the frontal lobe | Last EEG at the age of 13 years was normal | Hypodontia, teeth hypoplasia, widely spaced teeth, gingival hyperplasia | Global developmental delay, ID, muscle weakness, motor difficulties, hypotonia, Speech problems, Exotropia, muscle atrophy, microcephaly |
|  |  |  | M/12 | 1 day | NA | At the age of 8 years old, the EEG was normal | Hypodontia, teeth hypoplasia, widely spaced teeth, gingival hyperplasia | Global developmental delay, ID, speech problems, feeding difficulties |
| Alsemari et al.,2024 | F1 | c.291_296dupCGTGGC  (p.Ala99_Val100dup)  Hom | M/17  F/15  M/18  M/20 | Neonata | Brain volume loss and nonspecific signal abnormality of the bilateral hemispheric white matter | Epileptic discharges over the left anterior frontal lobes. | Teeth anomalies | Developmental delay, spasticity, wheelchair bound and spoke only a few words. |
|  | F2 | c.231+2T>G  Hom | M/15  M/19 | Neonatal | Normal | multifocal spikes | Dental anomaly | Profound global developmental delay, spasticity |
|  | F3 | c.691dubG  (p. Val231Glyfs*13)  Hom | F/6 | Neonatal | Persistent right posteroinferior cortical cerebellar T2/fluid-attenuated inversion recovery hyperintensity with no associated diffusion restriction | Nonspecific cerebral dysfunction | Teeth hypoplasia | Profound global developmental delay, spastic quadriparesis |
|  | F4 | c.1227dupC  (p. lle410Hisfs*13)  Hom | F/7 |  | Mild thinning of the corpus callosum, and patchy relatively symmetric T2/fluid-attenuated inversion recovery-hyperintense signal is noted in the bilateral hemispheric white matter | Spikes, over the left occipitotemporal region. | Teeth anomaly | Global developmental delay |

Het: heterozygous, Hom: homozygous,NA: Not Available,ID=intellectual disability, EE=Epileptic encephalopathy, MRI: magnetic resonance imaging, EEG:electroencephalogram,PWML=punctate white matter lesions.
